# A 74-Year-Old Man with Massive Ascites: A Case Report of Yellow Nail Syndrome

**DOI:** 10.3390/reports8040219

**Published:** 2025-10-30

**Authors:** Iliana Stamatiou, Melina Ntoga, Christos Karagiannis, Pipitsa N. Valsamaki, Dimitrios Papazoglou, Petros Rafailidis

**Affiliations:** 1 Second University Department of Internal Medicine, Democritus University of Thrace, 68100 Alexandroupolis, Greece; iliana_o6@hotmail.com (I.S.); melinant22@gmail.com (M.N.); chriskaragiannis7@hotmail.com (C.K.); dpapazog@med.duth.gr (D.P.); 2 Nuclear Medicine Department, Democritus University of Thrace, 68100 Alexandroupolis, Greece; pivalsam@med.duth.gr

**Keywords:** yellow nail syndrome, lymphedema, chylous, ascites

## Abstract

**Background and clinical significance**: Lymphedema is a relatively common clinical manifestation in patients and has a broad differential diagnosis, the main concern being the exclusion of malignancy. However, a rare constellation of lymphedema with systemic features and no underlying malignancy is yellow nail syndrome (YNS). YNS is a lymphatic abnormality, characterized by a triad of yellow nails, primary lymphedema and respiratory manifestations. **Case presentation**: Here, we report a 74-year-old male patient who presented to us with massive chylous ascites, cough, yellow nails and recurrent bilateral leg edema. During the last 10 years, he had thrice undergone thoracocentesis, which revealed chylous pleural effusion, although there was no documented diagnosis of yellow nail syndrome. We pursued a thorough work-up to rule out underlying cirrhosis and malignancy (the main causes of chylous ascites). There are only few cases of yellow nail syndrome reported in the literature with chylous ascites as a manifestation of YNS. **Conclusions**: The co-existence of chylous ascites with the classical triad of pleural effusion, lymphedema and yellow nail changes in the same patient has to be included in the diagnostic process to differentiate this entity from liver cirrhosis and solid or hematological cancer.

## 1. Introduction and Clinical Significance

Yellow nail syndrome (YNS) is a very rare disease with an estimated prevalence as low as 1/1,000,000 in the general population [1]. It was first described by Heller in the 1920s and, as of now, approximately 400 cases have been described in the overall literature [2]. In general, the syndrome usually develops after the fifth decade of life and affects both genders equally [2]. The main diagnostic tool for YNS is detailed clinical examination and comprehensive history as the classic triad of dystrophic nails, lymphedema, and respiratory tract disease remains crucial for the disease definition. Approximately 27% of patients have been reported to have all three signs/symptoms simultaneously, while in the remaining cases, a solitary symptom may persist for years, delaying the diagnosis. This case presentation highlights the presence of massive chylous ascites as a main presenting feature of YNS.

## 2. Case Presentation

A 74-year-old man was referred to our department complaining of bilateral leg edema along with refractory abdominal distention for 6 weeks. The patient reported no clinical improvement after a short course of diuretic therapy (furosemide 160 mg/d, eplerenone 25 mg/d, indapamide 5 mg/d) by his cardiologist; at that time, he presented with worsening dyspnea on exertion, nonproductive cough, and weight gain over the preceding 2 months.

He had a past medical history of hypertension, Chronic Obstructive Pulmonay Disease (60 pack/years), myasthenia Gravis, and benign prostatic hyperplasia. Approximately 1 year before this presentation, he had undergone surveillance endoscopy of the upper and lower gastrointestinal tract with normal findings. Also, he denied alcohol consumption, illicit drug use, or significant family history of any illness. Of note, the patient was hospitalized three times during the last 10 years due to recurrent episodes of pleural effusion. Thoracentesis revealed exudative, chylous, lymphocyte-predominant pleural effusion twice and transudative once. Furosemide at a daily dose of 40 mg was administered, while attending regular follow-up.

**Figure 1:**
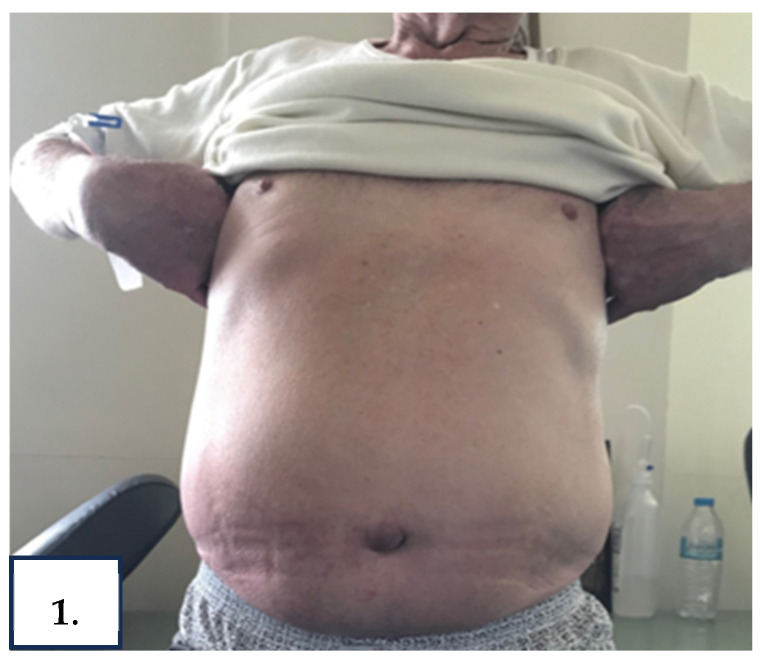
Abdominal distension due to massive chylous ascites.

**Figure 2:**
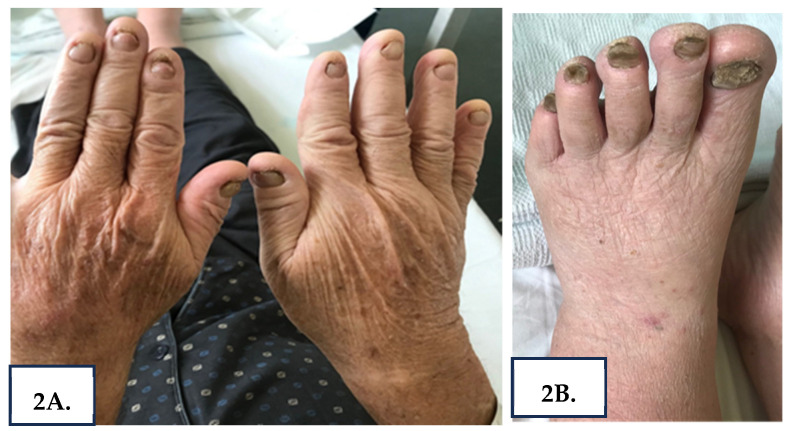
(**A**,**B**) Yellow discoloration and thickening of fingernails and toenails, with ridging and distal onycholysis—hallmark features of YNS.

Upon admission, the patient’s BMI was 30.5 kg/m^2^. He was afebrile and normotensive with mild tachycardia and good pulse volume. The thorough systemic physical examination showed diminished breath sounds in the right middle and lower lung fields, painless, non-pitting leg edema, anasarca, ascites (grade 3) (Figure 1), and thickening of fingernails and toenails with associated yellow discoloration (Figure 2A,B).

Laboratory testing was remarkable for hypoalbuminemia (3.4 g/dL) with normal urine albumin-to-creatinine ratio (UACR < 30 mg/g). Electrolytes, creatinine, albumin, liver function tests, and coagulation parameters were normal (Table 1). Serum NT-pro brain natriuretic peptide levels was also normal.

**Figure 3:**
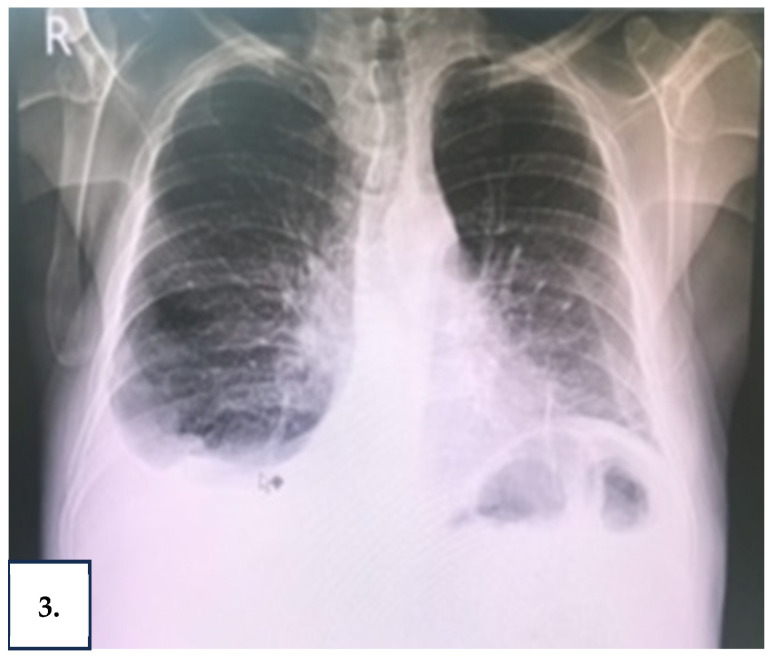
Chest radiograph showing a large right-sided pleural effusion with ipsilateral basal atelectasis, a common pulmonary manifestation of YNS.

Chest X-ray showed a right pleural effusion, pulmonary congestion, and atelectasis of the right lung base (Figure 3). Ultrasound of the abdomen was significant for a large amount of ascites.

**Figure 4:**
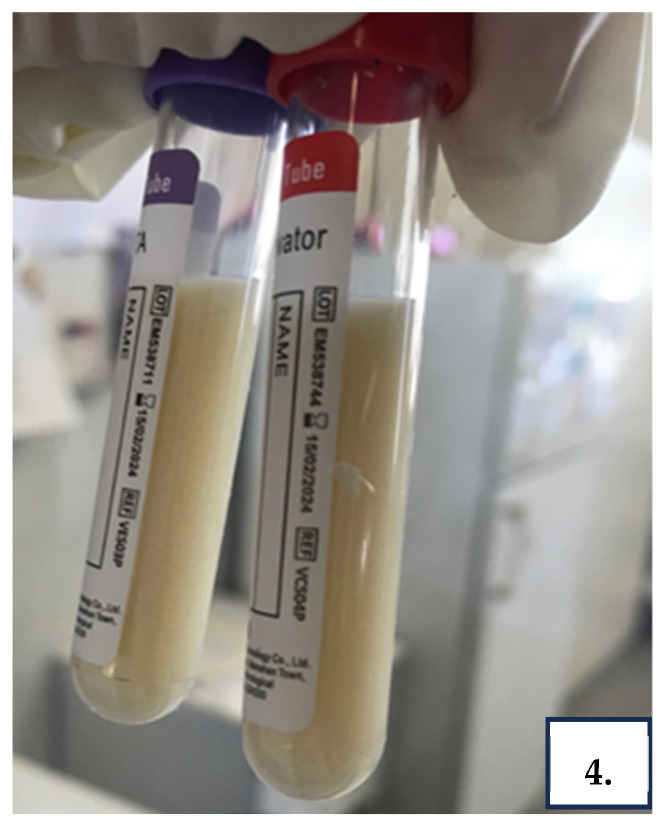
Milky-white chylous ascitic fluid obtained during diagnostic paracentesis, confirming the presence of triglyceride-rich effusion consistent with chylous ascites.

Then, an abdominal diagnostic and therapeutic paracentesis was performed and 5000 mL of milky-white ascitic fluid was aspirated (Figure 4). Routine analysis of ascitic fluid revealed that it was chylous ascites (Triglycerides 5010 mg/dL) with cell count 574/mm^3^ (lymphocyte predominant, 80%) and exudative with serum–ascitic albumin gradient 0.1; total protein 3.9 g/dL. Ascitic fluid culture for bacteria including *Mycobacterium tuberculosis* (MTB) was negative, as was molecular testing of ascitic fluid for MTB. Cytological examination of the ascitic fluid for malignancy was also negative.

**Figure 5:**
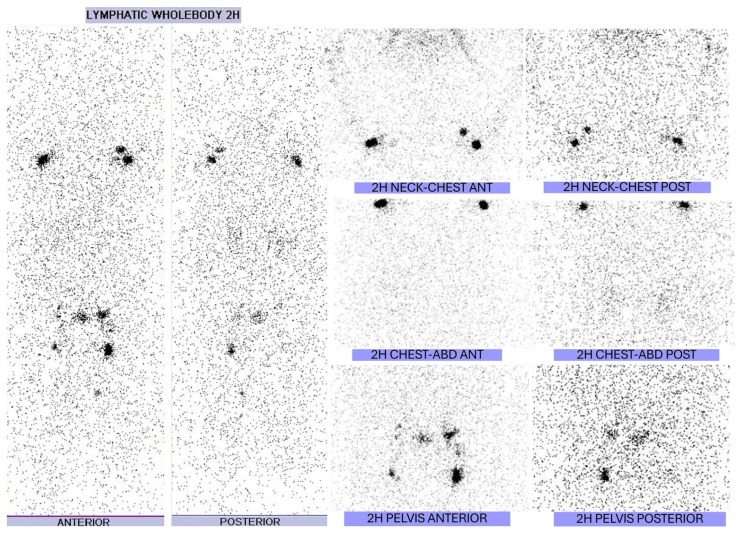
Radionuclide Lymphangiography revealing attenuation of periaortic and right pelvic lymph nodes, as well as of liver delineation, insinuating incomplete obstruction.

Subsequently, a comprehensive investigation was conducted to determine the cause of the chylous ascites. Serological markers of autoimmune diseases, as well as virological profile for hepatitis, did not reveal abnormal findings. His transthoracic echocardiogram ruled out a cardiac cause of ascites, as cardiac function, chamber diameters, and cardiac valves were found to be normal. A whole-body contrast-enhanced computed tomography scan showed bilateral pleural effusions, ascites and a dilated cisterna chyli, while there was neither evidence of lymphadenopathy nor of any abnormal mass; in addition, the size and morphology of the liver and spleen were normal. Furthermore, a whole-body radionuclide lymphangiography revealed attenuation of periaortic and right pelvic lymph nodes and of liver attenuation insinuating incomplete lymphatic obstruction. (Figure 5). In view of dystrophic nail lesions, a nail biopsy was performed that confirmed absence of onychomycosis. Based on the clinical manifestations and the investigations a diagnosis of YNS with chylous ascites was made.

Initially, administration of diuretics and a low-fat diet were planned during hospitalization. After discharge, the dosage of furosemide was increased to 80 mg/d and eplerenone to 50 mg/d. Four months after discharge, massive chylous ascites recurred and was aspirated twice. Eplerenone was titrated at 100 mg/d, Medium Chain Triglycerides (MCT) oil supplements were added and the patient responded very well.

## 3. Discussion

We present herein a patient with YNS and massive chylous ascites. Our patient exhibited the complete classical triad of yellow nails, lymphedema, and pleural involvement, in addition to the rare finding of chylous ascites. Chylous ascites refers to the pathological accumulation of triglyceride-rich lymphatic fluid within the peritoneal cavity, typically defined by ascitic triglyceride levels exceeding 200 mg/dL [3]. Most cases of chylous ascites are secondary due to cirrhosis, lymphoproliferative diseases, malignancies, drugs and infections like tuberculosis and filariasis. On the other hand, primary chylous ascites, whose underlying pathogenesis remains elusive, usually signifies lymphangiectasia with or without concomitant obstruction of lymphatic flow [4]. Our work-up did not identify any other association of the chylous ascites such as liver cirrhosis or malignancy. Given the presence of incomplete obstruction on radionuclide lymphangiography, without lymphadenopathy or presence of mass on whole-body Computed Tomography scan, the coexistence of chylothorax and chylous ascites in our patient likely reflects functional impairment or micro-trauma of the thoracic duct, resulting in chyle leakage into the pleural and peritoneal cavities—an established but rare mechanism in YNS. We identified in the literature only a few patients with YNS and associated chylous ascites [5,6,7,8,9,10,11,12,13].

The nail discoloration of our patient is part of a broader spectrum of nail deformities that occur in YNS. Yellow discoloration is the most prominent and commonly reported finding of the YNS triad. Clinicians should have a high level of suspicion because discoloration can vary from xanthonychia (pale yellow color) to dark green, and is thought to be secondary to oxidation of nail lipids [14]. Additional deformities include scleronychia, hyperkeratosis, and onycholysis (distal separation of the nail plate-coccidia), and complete nail loss occurs occasionally [15]. Moreover, it is important to recognize that the characteristic yellow-green nail discoloration may also occur in conditions other than YNS. Fungal infections (Candida, Aspergillus, or dermatophytes) and certain medications such as D-penicillamine have been implicated in similar presentations [16]. Therefore, a nail biopsy and histopathological examination is essential to exclude onychomycosis and confirm the diagnosis.

Another important aspect of our patient’s clinical characteristics was the recurrent and sustained pulmonary manifestations. Respiratory signs or symptoms in YNS, which occur in 56–71% of patients [2], vary from chronic cough, the most common pulmonary symptom, to intermittent pleural effusions arousing in 14–46% of them [17]. Pleural effusions can be chylous or non-chylous, while chylothorax accounts for 20% to 30% of all cases [2]. Our findings about characteristics of pleural effusions in YNS are directly in line with previous reports. This suggests that most pleural effusions tend to be bilateral, lymphocytic exudative, while only 5% have lymphocytic transudative [18]. In regard to the appearance of the fluid, 75% appear to be serous, 22% milky (chylothorax) and 3.5% purulent (empyema) [19]. Regarding the usefulness of histological examination of pleural fluid does not remarkably contribute to the diagnosis of YNS, revealing in most cases chronic fibrotic pleurisy with lymphocyte predominance. In view of therapeutic management, respiratory manifestations are usually treated according to the severity of symptoms; either conservatively with postural drainage physiotherapy or with thoracentesis and pleurodesis being sufficient to manage recurrent cases [20]. In our patient, invasive diagnostic procedures, including pleuroscopy or pleural biopsy, were not pursued given the non-malignant and recurrent nature of the effusions, the absence of pleural thickening on imaging, and the patient’s stable clinical status.

In the literature, lymphedema in YNS has an occurrence of 30% to 80% with no clinically apparent difference from primary lymphedema [2]. A hallmark clinical sign of primary lymphedema is Stemmer’s sign, which indicates weakness to pinch the skin on the dorsum or base of the second toe and is associated with underlying skin fibrosis [8]. Primary lymphedema is a chronic disease due to an increased volume of lymph accumulation and fibrosis resulting from excess fibroblast stimulation [21]. Lymphedema in YNS, as was the case in our patient, is usually non-pitting, painless, symmetrical and typically involves lower extremities [2].

In the field of management of YNS, several conservative treatment regimens were proposed, but they have focused on controlling the triad symptoms separately. As outlined in previous reviews, YNS mostly follows a wax and wane course of symptoms with a tendency towards spontaneous improvement [20].

Even if yellow nail discoloration is not caused by fungal infection, triazole regimens were used on a regular basis. Administration of itraconazole, at 400 mg weekly for 6–12 months in combination with oral vitamin E, at a dosage of 1000–1200 IU/day [22], and topical steroids such as triamcinolone acetonide [23] has been proposed with ambiguous results.

Management of pulmonary manifestations constitutes a major challenge due to their propensity for recurrence. Symptomatic treatments are strongly recommended. This approach is divided into two phases. At first, conservative measures such as chest physiotherapy, postural drainage, low-dose antibiotic prophylaxis, vaccinations against influenza and pneumococci are used to help patients for recurrent exacerbation [11]. Octreotide and lanreotide, somatostatin analogs, reduce intestinal lipid absorption and may improve lymphatic flow in individual cases of both chylous and non-chylous YNS pleural effusions. Octreotide has been initially given subcutaneously (0.5 mg twice daily), followed by the long-acting formulation (30 mg once/month) with or without dosage tapering as the mainstay of chylothorax treatment [24,25]. On the other side, for recurrent or large pleural effusions, surgical intervention with methods like pleurectomy, pleurodesis, and placement of pleural-peritoneal shunts has been reported as the most common approach with partial or complete response [9,11]. The same therapeutic approach is followed in cases of YNS chylous and non-chylous ascites as well, while peritoneovenous shunt has been advocated for refractory ascites cases.

For lymphedema, treatment focuses on conservative measures to reduce exacerbations and risk of infection and improve quality of life. The literature suggests skincare, manual lymphatic drainage, massage, high-pressure elastic garments, diuretics, and a possible therapeutic role for somatostatin analogues in refractory and recurrent cases [26].

In our report, the patient demonstrated a favorable clinical response to dietary modification with MCT oil supplementation and optimization of diuretic therapy through gradual titration of furosemide and eplerenone. This combined approach resulted in sustained control of chylous ascites and overall clinical stability. At the 19-month follow-up, the patient remained asymptomatic, with only intermittent therapeutic paracentesis required every 4–6 months.

## 4. Conclusions

YNS is a very rare disorder associated with yellow nail discoloration, pulmonary manifestations, and lymphedema. This report illustrates an uncommon presentation with massive chylous ascites, emphasizing the importance of considering YNS in the differential diagnosis of unexplained chylous effusions. Despite the rarity of this association, timely recognition is essential to avoid unnecessary invasive investigations and to guide appropriate conservative management. Further research is warranted to elucidate the underlying lymphatic mechanisms and optimize therapeutic strategies for this under-recognized condition. We hope that our case provides a stimulus for consideration of YNS as a cause of chylous ascites.

## Figures and Tables

**Table 1:** Relevant laboratory values.

Laboratory Parameter	Reference Range	Patient’s Values				
		D0	D1	D3	D7 ^†^	D45 ^‡^
White cell count (× 10^9^/L)	4.0–10.0	7.04	5.92	8.67	6.40	7.04
Hemoglobin (g/L)	12.0–16.0	16.1	13.7	15.0	13.9	14.2
Platelet count (× 10^9^/L)	150–400	367	333	361	253	313
International normalized ratio (INR)	0.8–1.2	1.15	1.09	1.16	1.13	1.10
Albumin (g/dL)	3.5–5.2	3.5	3.5	3.5	3.4	3.8
Total Protein (g/dL)	6.2–8.5	6.3	6.2	5.8	5.8	6.2
Total Bilirubin (mg/dL)	0.3–1.2	0.5	0.45	0.5	0.4	0.5
Alanine aminotransferase (U/L)	0–35	29	27	25	22	25
Aspartate aminotransferase (U/L)	0–40	38	31	35	48	40
Alkaline phosphatase (U/L)	30–120	66	57	66	48	51
Lactate dehydrogenase (U/L)	140–246	181	104	138	216	150
Urea (mg/dL)	15–45	56	52	49	66	71
Creatinine (mg/dL)	0.6–1.1	1.0	0.9	1.1	1.1	1.4
Potassium (mmol/lt)	3.5–5.1	3.8	3.4	3.8	4.1	4.1
Natrium (mmol/lt)	136–146	134	134	136	134	131
C–reactive protein (mg/dL)	<0.5	1.45	1.02	1.05	0.66	0.5
Urine, protein		-			-	
NT–pro BNP * (pg/mL)	<125	52.1				
Ascitic Fluid Analysis						
Gross appearance	clear, light yellow		Cloudy, yellow			Cloudy, white
Total Protein (g/L)	<4.1		3.9			3.8
Lactate dehydrogenase (U/L)	>Upper limit of serum reference interval		<50			<50
Triglyceride Level (mg/dL)	<200		5010			3050
SAAG ** (g/dL)	</≥1.1		0.1			0.2
Cell counts (cells/µL)	<500		574			1000
Neutrophils, N (%)	<25		20			23
Bacterial culture	Negative		Negative			Negative
M. Tuberculosis complex RT–PCR ***	Negative		Negative			
Cytology	Normal		Normal			

* NT-pro brain natriuretic peptide; ** serum-ascites albumin gradient; *** Mycobacterium Tuberculosis complex Real Time-Polymerase Chain Reaction; ^†^ Day of hospital discharge. ^‡^ Day of 1st re-assessment after discharge.

## Data Availability

The original contributions presented in this study are included in the article. Further inquiries can be directed to the corresponding author.
